# The exposome impact on hair health: non-pharmacological management. Part II^⋆^^☆^

**DOI:** 10.1016/j.abd.2024.08.006

**Published:** 2025-01-14

**Authors:** Stephano Cedirian, Ludmila Prudkin, Juan Antonio Santana, Jaime Piquero-Casals, David Saceda-Corralo, Bianca Maria Piraccini

**Affiliations:** a Dermatology Unit, IRCCS Azienda Ospedaliero-Universitaria di Bologna, Policlinico S. Orsola-Malpighi, Bologna, Italy; b Department of Medical and Surgical Sciences, Alma Mater Studiorum University of Bologna, Italy; c Innovation and Development, ISDIN, Barcelona, Spain; d Department of Dermatology, Clínica Dermatológica Multidisciplinar Dermik, Barcelona, Spain; e Servicio de Dermatología, Hospital Universitario Ramón y Cajal, Madrid, Spain; f Trichology Unit, Grupo de Dermatología Pedro Jaén, Madrid, Spain

**Keywords:** Alopecia, Cosmetics, Exposome

## Abstract

Hair holds a significance that surpasses mere aesthetics, as it plays a pivotal role in our social interactions and contributes significantly to the definition of our self-esteem. Central to this understanding is the concept of the exposome, which encompasses intrinsic elements like genetics and physiological changes, as well as extrinsic factors such as UV radiation, pollution, lifestyle choices, and chemical treatments. These factors may significantly impact hair health and hair aging. Expanding upon the groundwork laid by the first part of this research (Cedirian et al., 2024), this study aims to deepen our understanding of exposome influence on hair. Specifically, through a narrative review of current literature, this second part endeavors to provide non-pharmacological treatment solutions and effective strategies to mitigate the negative impact of the exposome on hair health.

## Introduction

Hair bears importance beyond its visual appeal, serving as a crucial element in our social interactions and contributing significantly to our self-esteem.^1^ Hair health is profoundly influenced by exposomal factors, which encompass all exposures individuals encounter from birth to death, including external and internal elements, as well as the body's responses to them.^2^ Continuous exposure to such elements can compromise hair health, resulting in issues such as thinning, breakage, weathering, and premature aging. Key exposomal elements impacting hair health include nutrition, drugs, stress and tobacco consumption as well as environmental conditions such as UV exposure, pollution and humidity among others.^3^, ^4^, ^5^, ^6^, ^7^, ^8^ Recognizing the interplay between the exposome and hair health is crucial for devising effective strategies to mitigate their adverse effects. The intricate interplay between nutritional supplements and cosmetic solutions in addressing exposomal impacts on hair health and aging has emerged as a focal point in contemporary clinical studies and strategies.^9^, ^10^, ^11^, ^12^ This comprehensive review delves into the diverse therapeutic approaches, ranging from micronutrients like biotin, iron, vitamins D and E, and zinc, to non-pharmacological androgen modulators such as pumpkin seed oil and *Serenoa repens*. Additionally, the article evaluates the potential of cosmetic interventions, examining the effectiveness of substances like capsaicin, horsetail, and ashwagandha, while shedding light on other topical applications and oils. The primary aim of this paper is to fulfill the requirement for effective non-pharmacological therapeutic approaches by conducting a thorough review of existing literature; specifically, the goal is to provide comprehensive guidance on mitigating the impact of the exposome on hair health and aging.

## Non-pharmacological treatment strategies

Addressing the exposomal impact on hair health and hair aging represents one of the pivotal steps clinicians should prioritize. The available literature is rich with diverse approaches to hair diseases, spanning from nutritional supplements to cosmetic solutions.

### Nutritional supplements

Currently, dietary supplements are a prominent choice when it comes to dealing with hair health; however, they are categorized as food. Indeed, the Food and Drug Administration (FDA) does not govern their safety or effectiveness.^9^

Biotin, also known as vitamin B7, is contained in different nutritional supplements as it serves as a crucial cofactor for numerous enzymes involved in various metabolic reactions, including those related to glucose, fatty acids, and amino acids. Furthermore, biotin plays a significant role in gene regulation and protein synthesis, particularly within sheep hair follicles (HFs).^9^, ^10^ Although biotin deficiency is relatively rare in Western countries due to the consumption of well-balanced diets, certain individuals are at higher risk. Factors contributing to deficiency include congenital holocarboxylase synthetase or biotinidase deficiency, pregnancy, long-standing breastfeeding, certain medication intake (such as valproic acid and isotretinoin), and excessive consumption of raw egg whites. Despite its popularity as an ingredient in various supplements, particularly for treating onychoschizia, evidence supporting biotin's efficacy in randomized controlled trials, especially for hair loss treatment, remains scarce.^9^, ^10^ Moreover, high biotin intake can lead to significant interference with laboratory tests, particularly those utilizing biotin-streptavidin immunoassays. This interference can yield inaccurate results, as demonstrated by a 2017 FDA safety communication citing a patient's cardiac-related death due to falsely low troponin levels caused by biotin interference. Consequently, caution is advised when interpreting laboratory results, especially for individuals consuming high-dose biotin supplements.^9^, ^10^ Narda and colleagues illustrated the effectiveness of a dietary supplement comprising L-cystine, *Serenoa repens* extract, and biotin. In their research, men with androgenetic alopecia (AGA) exhibited a 23.4% rise in the anagen/telogen ratio compared to the initial measurement after 6-months of treatment (p < 0.01), whereas the group of women with telogen effluvium (TE) reported an important improvement in results of the hair pull test. Conversely, no notable difference was observed in the placebo cohort. Participants receiving the supplement reported increased hair volume (p < 0.01), while those in the active group noted improved hair appearance (p < 0.05). Quality of life and efficacy exhibited insignificant enhancement in questionnaire ratings within the test group compared to the placebo.^11^ However, the effect of biotin on the hair shaft remains uncertain, and its supplementation alone does not appear promising in enhancing hair growth speed.^13^

Regarding iron, its deficiency is widespread, affecting populations such as pregnant women, infants, vegetarians and individuals with conditions like heart failure. While iron deficiency has been linked to hair loss, research findings vary. Several studies have indicated associations between iron deficiency and TE, alopecia areata (AA) and AGA, suggesting a potential link between low iron levels and hair loss.^10^, ^14^, ^15^, ^16^ For instance, a recent study suggested that subjects with telogen effluvium who had higher baseline levels of ferritin felt more satisfied with iron supplementation. This indicates that higher levels of ferritin may positively influence the hair cycle.^16^ Although it remains controversial, levels of ferritin above 40 mg/mL are supposed to be enough to avoid hair shedding.^17^ Iron supplementation should be carefully managed due to potential adverse effects and interactions with medications like levothyroxine and levodopa.^10^

Among other micronutrients, Vitamin D regulates the expression of genes involved in HF cycling and growth. Deficiency may disrupt the normal hair growth cycle, leading to hair thinning and shedding.^18^, ^19^, ^20^ Vitamin D deficiency is associated with autoimmune diseases, including AA, where the immune system attacks HFs. Optimal vitamin D levels have shown promise in regulating immune responses, yet further research is essential to solidify this correlation.^10^, ^21^ However, there are no studies demonstrating improvement in hair loss with vitamin D supplementation.

Vitamin E's antioxidant properties protect HFs from oxidative stress and weathering caused by free radicals. It promotes scalp health and supports hair growth by maintaining the integrity of cell membranes; indeed, vitamin E supplementation may stimulate hair growth by promoting blood circulation to the scalp and enhancing nutrient delivery to HFs.^9^, ^10^, ^22^

Vitamins could potentially play a significant role in managing hair loss. However, considering the widespread use of multivitamins with low vitamin concentrations, it is important to clearly state that there is no evidence supporting the effectiveness of this practice.

Finally, zinc is another micronutrient that serves as a cofactor for numerous enzymes involved in DNA synthesis, cell proliferation, and tissue repair, processes critical for hair growth and maintenance. Zinc acts as an antioxidant, protecting HFs from oxidative damage and inflammation, which can contribute to hair loss. Zinc plays a role in regulating steroid hormone levels, including those involved in hair growth and follicle health, and it is an essential cofactor for vitamin D activity. Imbalances in zinc levels may disrupt hormonal signaling pathways, leading to hair loss.^9^, ^10^, ^23^, ^24^ Studies have shown that patients with hair conditions like AA and TE tend to have lower serum zinc levels compared to healthy individuals, particularly in AA and TE groups.^25^, ^26^, ^27^

Among nutritional supplements, androgen modulators have been proposed as well. Pumpkin seed oil (PSO), also known as *Cucurbita pepo*, has been studied for its potential therapeutic effects, including its ability to inhibit 5-alpha reductase, which converts testosterone to dihydrotestosterone (DHT), a hormone implicated in hair loss. PSO is rich in essential nutrients like zinc, iron, potassium, selenium, magnesium, and calcium, making it a promising candidate for addressing various health issues.^28^ In a randomized, double-blind, placebo-controlled trial, patients with AGA received oral PSO for 24 weeks. The study showed a statistically significant increase in hair count in the group receiving PSO, indicating its potential efficacy in treating hair loss. However, the exact impact of PSO remains somewhat uncertain due to the inclusion of additional ingredients in the supplement. Furthermore, the trial specifically focused on AGA, necessitating further research to assess PSO's effectiveness across different types of hair loss conditions.^9^, ^12^

*Serenoa repens* (also known as saw palmetto extract), another natural inhibitor of 5-alpha reductase, has also been investigated for its potential in treating AGA.^29^ A randomized clinical trial comparing *Serenoa repens* to finasteride, a conventional treatment for hair loss, has shown promising results. In a 2-year trial, palmetto demonstrated efficacy in increasing hair growth, though to a lesser extent compared to finasteride. Another study found significant improvement in patients with AGA after using *Serenoa repens* for 18 weeks.^30^ Despite these positive findings, the evidence supporting the use of saw palmetto in treating hair loss remains limited, particularly regarding its effectiveness in other types of hair loss conditions beyond AGA. Further research is needed to validate the results and determine the appropriate dosage and duration of treatment for *Serenoa repens* in addressing various forms of hair loss.^9^, ^31^

Horsetail (*Equisetum arvense*), an herbaceous plant, has a long history in traditional medicine due to its anti-inflammatory, antioxidant, and antimicrobial properties. It contains silicon, which takes part in collagen synthesis as orthosilic acid, potentially supporting hair health.^32^, ^33^ Clinical trials have shown promising results regarding its effects on hair and nail brittleness, as well as hair thickness and elasticity. Nonetheless, further trials are needed to confirm these findings and determine the optimal usage of horsetail for promoting skin and hair health.^34^, ^35^ Regarding horsetail, Barel et al. conducted a 20-week randomized, placebo-controlled, double-blind study to assess the effects of oral choline-stabilized orthosilicic acid (10 mg/day) on hair and nails in females with photoaged skin. The study reported significantly lower scores in hair and nail brittleness in the active group compared to placebo.^33^ Wickett et al. performed a 9 month randomized, placebo-controlled, double-blind study to evaluate the effects of oral choline-stabilized orthosilicic acid (10 mg/day) on hair morphology and tensile strength in women with fine hair. The active group showed a significant change in cross-sectional area, indicating thicker hair compared to the placebo group.^34^

In literature, it is possible to find other compounds explored for their different properties associated with hair health. Ashwagandha (*Withania somnifera*) is known for its antioxidant and adaptogen properties, which are central to Ayurvedic medicine. Studies suggest that ashwagandha may help reduce perceived stress and cortisol levels in the body, which are linked to hair loss.^36^ Additionally, there's a proposed mechanism suggesting that ashwagandha may enhance blood flow to the scalp, which could benefit hair growth.^37^ Further research is needed to validate its direct impact on hair growth.^12^

### Cosmetic solutions

An alternative approach to mitigating the exposomal effects on hair health and aging involves the application of cosmetic products. For example, literature has explored photoprotection of the scalp and hair as a means to mitigate the adverse effects of UV radiation. Chemicals applied topically for sun protection are widely used and offer convenient means to shield smooth skin from the immediate (sunburn) and long-term effects of UV radiation. However, their efficacy on the scalp with hair remains uncertain. Although there is extensive research on protecting hair from sun damage, there is a noticeable lack of data on safeguarding the hair-bearing scalp. Some studies suggest that certain substances like cinnamidopropyltrimonium chloride found in shampoos may offer protection against UV damage to hair.^38^ Additionally, solid lipid nanoparticles have been developed to carry UV blockers for both skin and hair protection. These nanoparticles not only carry UV blockers but also reflect and scatter UV radiation themselves.^37^, ^39^ Recent research has also delved into the impact of metals like copper on hair weathering under UV exposure.^40^, ^41^ This research offers insights into mitigating copper-related damage using chelating agents. For instance, certain studies have found that copper in water can accelerate UV-induced damage to hair, leading to protein degradation and the formation of specific marker peptides. Strategies to reduce copper levels in hair using chelating agents like N,N’-ethylenediamine disuccinic acid have been explored.^15^, ^40^, ^41^, ^42^

Melatonin, a multifunctional hormone, impacts various physiological processes, including those related to HFs. Babdjouni et al. uncovered 11 human studies featuring oral or topical melatonin utilization in individuals diagnosed with alopecia (2,267 patients; 1,140 M). Among these studies, eight observed favorable outcomes following the application of topical melatonin in individuals affected by AGA, with varying doses (topical 0.0033% melatonin, topical 0.1% melatonin, topical 25 mg melatonin in 200 mg lipid mixture and topical 25 mg melatonin with 250 μL lipid mixture).^43^, ^44^, ^45^, ^46^, ^47^, ^48^ The majority of the studies noted enhancements in scalp hair growth (n = 8), density (n = 4), and hair shaft thickness (n = 2) among melatonin users compared to controls. Effective topical melatonin doses were found to be 0.0033% or 0.1% solutions applied once daily for 90 to 180 days, as opposed to 1.5 mg twice-daily oral melatonin supplementation for 180 days. These pieces of evidence suggest that melatonin usage can support scalp hair growth, particularly in men with AGA.^48^ Furthermore, topical melatonin has shown promise in reducing UV-induced skin reactions, suggesting its potential use in combination with conventional sunscreens for enhanced protection of hair as well.^15^

Capsaicin, the compound responsible for the spicy flavor, is utilized topically to alleviate neuropathic and musculoskeletal pain.^49^ Studies have indicated a positive impact on hair growth, particularly in patients with alopecia. However, the evidence is still limited, and more research is necessary to establish its effectiveness as a treatment for hair loss.^50^ In the 12-week open-label trial conducted by Ehsani et al., topical capsaicin was compared to clobetasol ointment in patients with AA. The study revealed a statistically significant increase in vellus and non-cosmetic hair growth in individuals using capsaicin compared to clobetasol. However, there was no significant difference in cosmetically significant hairs.^50^

Among other topical alternatives, Yerram et al. explored the effects of Ashwagandha (*Withania somnifera*) root extract serum on hair health. They conducted a study to assess the efficacy and safety of Ashwagandha serum on hair health in healthy adults. In a double-blind, randomized trial, participants using Ashwagandha showed significant improvement in hair density, growth, thickness, and quality of life compared to those using a placebo. The study suggests that topical Ashwagandha could serve as a beneficial and safer treatment option for alopecia.^51^

The effects of mineral and vegetable oils on human hair have also been studied extensively. These oils, characterized by their hydrophobic nature, play a crucial role in protecting hair from weathering by reducing water absorption and preventing the penetration of harmful substances. Coconut oil stands out for its ability to reduce protein loss in both undamaged and damaged hair, attributed to its unique molecular structure that allows deep penetration.^52^, ^53^ On the other hand, mineral and sunflower oils primarily offer surface protection but may not penetrate the hair shaft effectively. The choice of oil can affect hair appearance and health, with thinner layers preferred for better absorption and reduced heaviness.^54^ Brazilian oils, including Brazilian nut and mineral oils, have shown promise in reducing split ends and improving combing force.^55^ Additionally, argan oil, rich in antioxidants, has gained popularity for its moisturizing properties, although further research is needed to fully understand its benefits for hair care.^56^

Lastly, conditioners that contain proteins are effective in enhancing hair health by temporarily repairing weathering, especially at split ends.^57^ Protein-based conditioners consist of small, hydrolyzed protein fragments, like amino acids or peptides, with molecular weights ranging from 1 to 10 kDa. These components can penetrate the hair shaft, bind to keratin, and restore lost proteins, ultimately enhancing hair strength and preventing further weathering.^58^, ^59^ Hydrolyzed proteins are sourced from various origins such as animal collagen, keratin, or placenta. The effectiveness of the conditioner depends more on the size of its particles and their ability to penetrate the hair shaft rather than the source of the protein.^57^, ^59^ Additionally, the duration of contact is crucial: leaving the conditioner on for a longer time allows the proteins to diffuse into the hair fibers more effectively. However, the conditioning effect of proteins is temporary as excess proteins are washed away during shampooing. Therefore, it's necessary to reapply the conditioner to maintain its effectiveness.^57^, ^60^

## Conclusion

As the pursuit of effective treatment strategies for hair health unfolds, the use of nutritional supplements and cosmetic solutions emerges as a dynamic pathway to address hair weathering induced by exposomal factors. While the potential efficacy of various treatments is evident in select studies, the field awaits further exploration, necessitating additional research to validate findings and establish comprehensive guidelines for clinicians. In conclusion, this article underscores the importance of a holistic approach to hair care, offering a nuanced understanding of the diverse strategies available to clinicians navigating the intricate landscape of exposomal effects on hair health and aging.

## Financial support

This study was not funded but the authors have been employed by ISDIN® to elaborate this project.

## Authors’ contributions

Stephano Cedirian: Performed the research, analyzed the data, wrote the paper, approved the final manuscript as submitted and agreed to be accountable for all aspects of the work.

Ludmila Prudkin: Performed the research, designed the research study, analyzed the data, wrote the paper, approved the final manuscript as submitted and agreed to be accountable for all aspects of the work.

Juan Antonio Santana: Performed the research, designed the research study, analyzed the data, wrote the paper, approved the final manuscript as submitted and agreed to be accountable for all aspects of the work.

Jaime Piquero-Casals: Performed the research, designed the research study, analyzed the data, wrote the paper, approved the final manuscript as submitted and agreed to be accountable for all aspects of the work.

David Saceda-Corralo: Performed the research, analyzed the data, wrote the paper, approved the final manuscript as submitted and agreed to be accountable for all aspects of the work.

Bianca Maria Piraccini: Performed the research, analyzed the data, wrote the paper, approved the final manuscript as submitted and agreed to be accountable for all aspects of the work.

## Conflicts of interest

The authors have been employed by ISDIN® to write this paper.
